# Towards identification of finger flexions using single channel surface electromyography – able bodied and amputee subjects

**DOI:** 10.1186/1743-0003-10-50

**Published:** 2013-06-07

**Authors:** Dinesh Kant Kumar, Sridhar Poosapadi Arjunan, Vijay Pal Singh

**Affiliations:** 1 Bio-signals Lab, School of Electrical and Computer Engineering, RMIT University, GPO Box 2476, Melbourne, Victoria 3001, Australia; 2 IP Australia, Canberra, Australia

## Abstract

**Background:**

This research has established a method for using single channel surface electromyogram (sEMG) recorded from the forearm to identify individual finger flexion. The technique uses the volume conduction properties of the tissues and uses the magnitude and density of the singularities in the signal as a measure of strength of the muscle activity.

**Methods:**

SEMG was recorded from the *flexor digitorum superficialis* muscle during four different finger flexions. Based on the volume conduction properties of the tissues, sEMG was decomposed into wavelet maxima and grouped into four groups based on their magnitude. The mean magnitude and the density of each group were the inputs to the twin support vector machines (TSVM). The algorithm was tested on 11 able-bodied and one trans-radial amputated volunteer to determine the accuracy, sensitivity and specificity. The system was also tested to determine inter-experimental variations and variations due to difference in the electrode location.

**Results:**

Accuracy and sensitivity of identification of finger actions from single channel sEMG signal was 93% and 94% for able-bodied and 81% and 84% for trans-radial amputated respectively, and there was only a small inter-experimental variation.

**Conclusions:**

Volume conduction properties based sEMG analysis provides a suitable basis for identifying finger flexions from single channel sEMG. The reported system requires supervised training and automatic classification.

## Background

Surface electromyogram (sEMG) is the non-invasive recording of the electrical activity of the muscle. It is closely related to muscle contraction and an indicator of the associated actions. For an amputee, sEMG of the residual muscles becomes an obvious choice for natural control of the prosthetic hand. This requires the classification of sEMG signals to identify the desired finger movements and obtain the command for controlling the prosthetic hand. Some of the earlier attempts to identify finger actions from sEMG were based on an estimate of the amplitude [1] and the rate of change of the sEMG [2]. More recent studies [3-19], have reported significant developments in the identification of movements for myoelectric control systems.

Researchers have reported success in the use of multiple channels sEMG recording for controlling the prosthetic hand [3-10]. Tenore et al. [7,10] have investigated the effectiveness of different configurations of electrode arrays (19 and 32) on the performance of the prosthetic control, both on able – bodied and trans-radial amputees. However, such systems are complex and the variation in electrode placement during sEMG recording can alter the signal and the outcomes significantly [17,20] making the technology unsuitable for self-administration by the user or their carer. There is also significant variation of sEMG magnitude and spectrum between different experiments due to a number of factors that cannot be controlled [12,21,22]. A single channel system that can reliably identify the finger actions and in which the location of electrodes is not critical, is highly desirable. Smith et al. [8] and Chen et al. [9] attempted to minimise the number of electrodes (using six to eight electrodes) to decode four different finger flexions. Another method reported by Englehart et al. [4] is based on dimensionality reduction using principal components analysis after wavelet decomposition. However, overlapping muscles and presence of noise and artefacts makes this a challenging task.

Many researchers have worked to decompose the sEMG signal into constituent MUAPs [23,24]. Pelvin and Zazula [25] reported the use of higher order statistics for decomposing the EMG signal into the fundamental components, the individual motor unit action potentials (MUAP). However, these techniques are based on the priori of shape and density of MUAP, making them unsuitable when there are multiple muscles because the shape of MUAP from different muscles can vary due to the difference in the transmission pathways. SEMG recordings integrate the electrical activity from all adjoining muscles and thus lack muscle selectivity. Further, low-level muscle activity, such as during finger flexion, makes the signal susceptible to noise and artefacts. Different choices of global features of the signal using advanced signal processing and pattern recognition techniques do not address these fundamental issues and such research can at best result in marginal improvement. While the systems reported in literature appear to be suitable for recognising gross movements, they are ineffective for complex movements such as wrist and finger movements [2] where a number of muscles are involved.

This paper reports an alternate method to estimate the relative strength of contraction of different muscles. It is based on the transmission properties of action potentials in body tissues [26-28] and on the assumption that the MUAP are sparse and cause singularities in the recordings. The MUAP arriving from one muscle were considered to have similar magnitudes, and those from other muscles have different magnitudes. Wavelet maxima were used to identify the singularities and cluster analysis was performed to determine the relative strength corresponding to different actions. The densities of the clusters of wavelet maxima were classified using a twin support vector machine (TSVM) to determine the associated finger flexion and the technique has been called Wavelet Maxima Density (WMD) technique.

## Methods

### Subjects

Eleven healthy subjects (7 male and 4 female; Mean age = 27.4 (±2.55) years; Mean weight = 68.7 (±3.56) kg; and Mean height = 169.3 (±6.2) cm) volunteered to participate in this study. The healthy participants exclusion criterion was; (i) no history of myo or neuropathology, and (ii) no evident abnormal motion restriction. The technique was tested on one amputee participant (Female; Age = 42 Years) who volunteered to take part in this study. Amputee volunteer had trans-radial one-third proximal amputation of the right forearm (with 9 cm long stump).

### SEMG recording procedures

**Figure 1:**
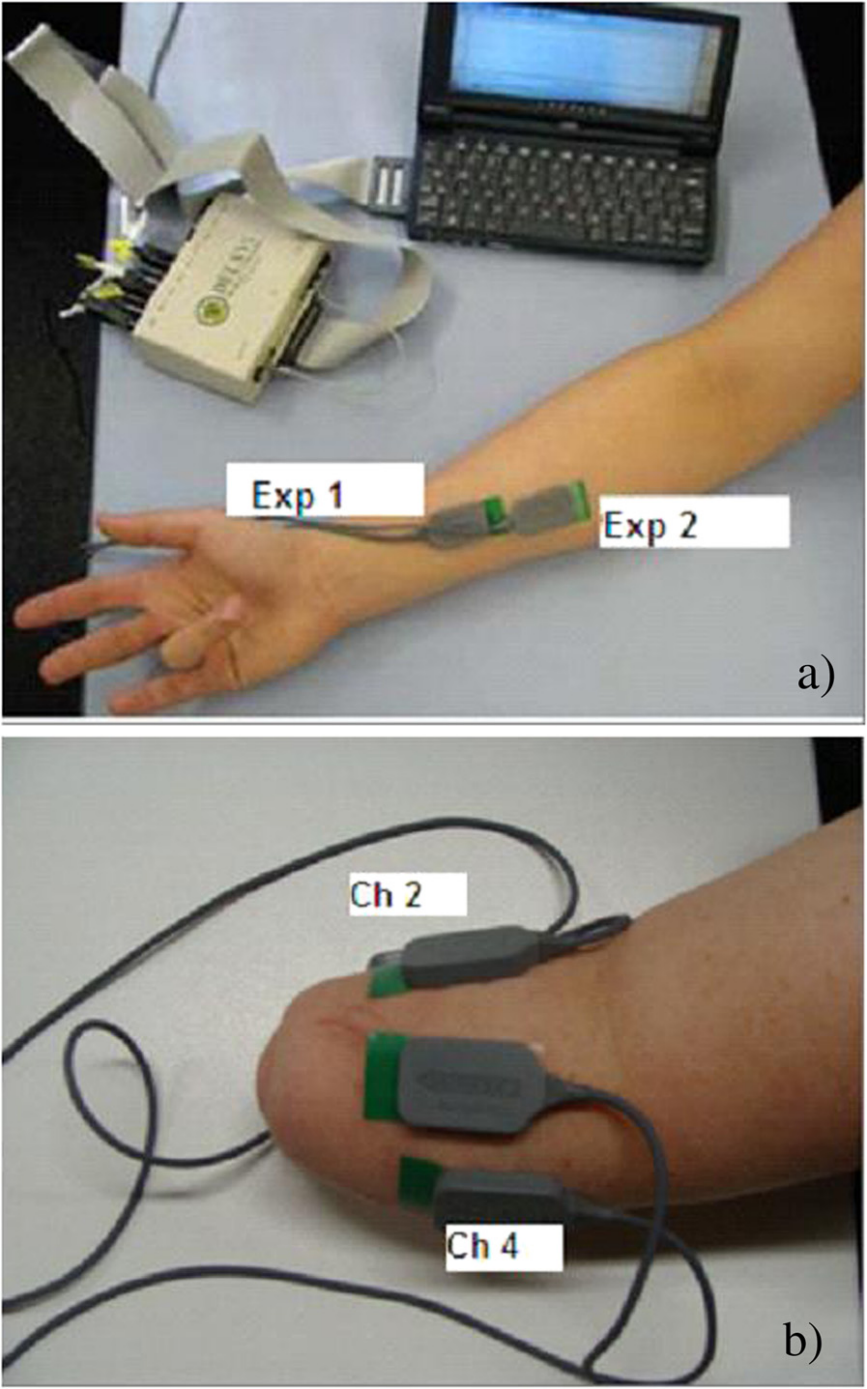
Electrode Placement a) at the distal (EXP1) and proximal end (EXP2) b) on the Trans- radial amputee.

Bipolar electrodes (DELSYS, Boston, MA, USA) were placed on the forearm (FDS) muscle for the healthy participants (Figure 1a) in accordance with standard procedures [29] to record surface electromyogram (sEMG). These are active electrodes, with the preamplifier and two electrodes built into a single unit. The electrodes are self-adhesive, and have two silver bars; each of 1 mm thickness, 10 mm length and the fixed inter-electrode distance of 10 mm. Electrolyte Gel (Sigma) were used on the electrodes prior to affixing them on the skin.

Experiments were repeated twice for the able-bodied volunteers, with electrodes placed on the proximal end in the first experiment- EXP1, and on the distal end for the second experiment – EXP2 (Figure 1a). This was done to determine the effect of variation in the electrode location on the outcome of the experiments. The ground electrode was placed on the volar aspect of the wrist. For the amputee participant, the electrodes were placed on the remaining stump of the participant as shown in Figure 1b.

DELSYS (Boston, MA, USA) sEMG acquisition system was used to record the signal. The system gain was 1000, CMRR was 92 dB, and bandwidth was 20–450 Hz, with 12 dB/ octave roll-off. The input impedance of the system was 115 Pico-farad in parallel with 1 K-ohm. The sampling rate of the system was 1024 samples/ second for each channel and the resolution were 16 bits/ sample. Prior to the placement of electrodes, the skin of the participant was prepared by shaving (if required) and exfoliation to remove dead skin. Skin was cleaned with 70% v/v alcohol swab to remove any oil or dust from the skin surface.

### Experimental protocol

**Figure 2:**
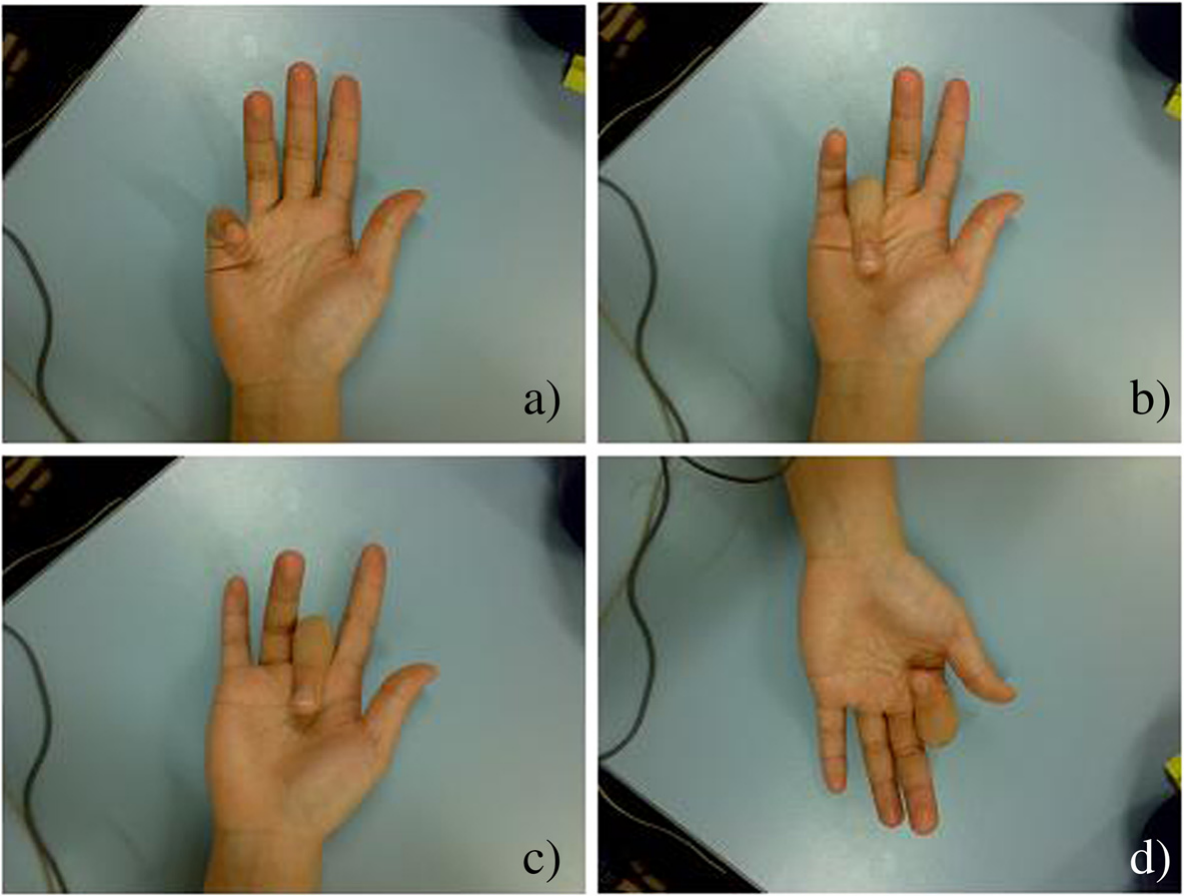
**Four finger flexions; (a) Little Finger; (b) Ring Finger; (c) Middle Finger; (d) Index Finger.** Proximal inter – phalangeal joint was flexed in these experiments.

Experiments were conducted where the sEMG was recorded while the participants performed four sets of generic finger flexion actions (Figure 2) labelled and described below.

• Background activity: All fingers resting.

• Class 1: Flexion of little (pinkie) finger

• Class 2: Flexion of ring finger

• Class 3: Flexion of middle finger

• Class 4: Flexion of index finger

These generic actions were selected for the following reasons:

• these actions will allow the user to control individual fingers in the recently available advanced robotic/ prosthetic hand [7,10,19] and utilise these devices to the maximum advantage.

• these actions can be used for communication and control commands that can be used for several different devices and applications.

The participants performed the flexion without any resistance and as was convenient and easily reproducible by them. SEMG was recorded through the experiment. The examiner gave on-screen and oral commands to the participant to perform the action without any fixed order of the fingers. Each flexion was maintained for about 7–8 seconds and was repeated twelve times. The experiments were repeated twice with changed electrode location.

The experimental protocol was approved by the RMIT University Human Ethics Committee and Alfred Health Ethics Committee and performed in accordance with the Declaration of Helsinki 1975, as revised in 2004. Prior to the recording, the participants were encouraged to familiarize themselves with the experimental protocol and with the equipment. For the experiment with the amputee, bilateral action training modality was performed [30]. The amputee subject performed the finger flexions with the healthy hand while attempting to repeat the same flexion with the phantom limb [30].

### Data analysis

#### Removal of noise and background activity

**Figure 3:**
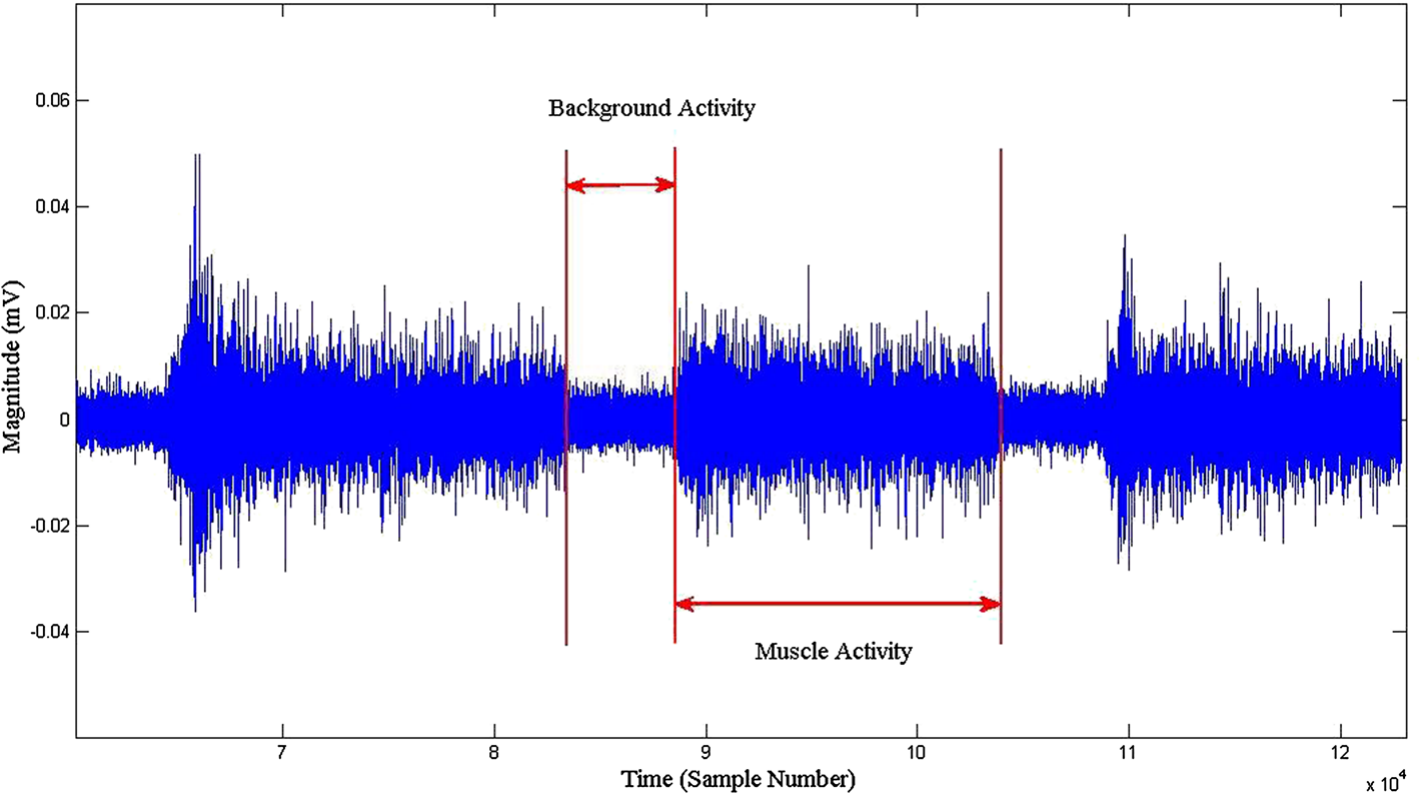
Example of the background and the muscle activity.

As a first step, the signal corresponding to class 1 for each subject on each day was normalised to the root mean square (RMS) of the recording of the same subject. The next step was the removal of the background activity from the signal (Figure 3). This becomes more challenging because when signal strength is low, the noise magnitude becomes comparable with the signal itself.

The pilot analysis of the recordings showed large inter-experimental variations in the spectrum of the background activity, making stationary spectral filtering unsuitable because of the variations in the noise characteristics [31]. For this reason, adaptive spectral subtraction filters were used to remove the background activity from the recorded signal. The template of the spectrum of the noise was obtained for each subject using a bandpass filter (4th order Butterworth, frequency 10 to 450 Hz) and averaging over 20 windows (>20).

#### Decomposing sEMG to obtain relative strength of contraction

The first step of the proposed method required the identification of the temporal location of the MUAPs. For this purpose, sEMG signal was decomposed using wavelet transform (bi-orthogonal wavelet ‘bior3.3’) and the maxima were identified based on the change of sign [32].

Local maxima, *Wf*(*s,x*_*n*_) can be described mathematically as follows [32]:

(1) $$\mathrm{Wf}\left(s,x_{n-1}\right)<\mathrm{Wf}\left(s,x_{n}\right)>\mathrm{Wf}\left(s,x_{n+1}\right)$$

Where *Wf*(*s,x*) is the wavelet transform of the function *f(x)* at a scale *s* and *n* = 2 to *N*-1, *N* is the total number of coefficients at any given scale *s*.

The wavelet maxima that were present in each of the scales and travelled from finest scale to coarsest scale were considered [33,34]. Other wavelet maxima were rejected as being random transients. The data of each flexion was segmented into 300 ms windows, and the magnitudes at the lowest (finest) scale of the accepted maxima were obtained. Based on the magnitude, the magnitude and number of maxima points of every windowed recording (density of peaks) corresponding to each flexion were the two dimensional representation of the recording. These were separated into four (number of different actions) groups using cluster analysis. The centroid and corresponding density of each cluster was determined for each time segment, and averaged for the duration of each flexion. This resulted in one value of the magnitude and the corresponding density of peaks for each action. This two dimensional feature set was used to train and test the system.

#### Classification

Twin support vector machines (TSVM) linear kernel classifier [35] was used to classify the features. The advantages of using TSVM is that it solves two related SVMs, one for each class, and generates two separate hyper planes without the assumption that patterns in each class arise from similar distributions. It allows the use of a different kernel for each class which can be separately optimized based on the data. This data dependent kernel optimization for each class is particularly useful in this study. For more details, please refer to [15,35].

The vectors corresponding to the four densities and four magnitudes were the input to the classifier. During the training phase the associated finger flexion was the target. After the system was trained, the system accuracy was validated using ten-fold cross – validation and tested using Type I error (Specificity) and Type II error (Sensitivity) [15]. The training dataset had 100 data points, and testing was done using 30 data points. The training data set and test data set was chosen randomly using random sub-sampling method. Ten-cross validation method was used to determine the accuracy, sensitivity and specificity of the system. Classification was performed on the data from the individual subject. The system was tested individually for the two experiments to determine the inter-experimental variations.

## Results

The average classification accuracy for able-bodied subjects is reported in Table 1 and for amputee subject in Table 2. The sensitivity and specificity results for the able bodied subjects are reported in Table 3 and for the amputee subject in Table 4.

**Table 1:** Mean classification accuracy (%) of different classes recorded from distal end (EXP1) and proximal end (EXP2) from able-bodied all subjects using WMD method

**Class**	**Distal end (EXP1)**	**Proximal end (EXP 2)**
	**Mean (±SD)%**	**Mean (±SD)%**
**Class 1**	92.84 (±4.47)	94.23 (±5.08)
**Class 2**	95.47 (±4.07)	95.96 (±3.08)
**Class 3**	92.10 (±5.98)	90.37 (±5.58)
**Class 4**	93.21 (±5.96)	89.06 (±8.17)
**Overall mean (±SD)**	**93.41 **(±1.54)	**92.4 **(±3.23)

**Table 2:** Mean classification accuracy (%) of different classes recorded from Ch 2 and Ch 4 from amputee subject using WMD method

**Class**	**Channel 2 mean (±SD)%**	**Channel 4 mean (±SD)%**
**Class 1**	96.67 (±9.44)	71.67 (±8.08)
**Class 2**	75 (±9.92)	62.42 (±8.74)
**Class 3**	66.67 (±10.67)	72.15 (±11.24)
**Class 4**	89.15 (±8.26)	92.14 (±7.52)
**Overall mean (±SD)**	**81.87** (±13.54)	**74.59 **(±12.52)

**Table 3:** Sensitivity (%) and specificity (%) for each class recorded from distal end (EXP1) and proximal end (EXP2) from able-bodied all subjects using WMD method

**Class**	**Distal end (EXP 1)**		**Proximal end (EXP 2)**	
	**Sensitivity mean (±SD)%**	**Specificity mean (±SD)%**	**Sensitivity mean (±SD)%**	**Specificity mean (±SD)%**
**Class 1**	92 (±0.05)	98 (±0.02)	94 (±0.07)	98 (±0.02)
**Class 2**	95 (±0.06)	97 (±0.03)	95 (±0.04)	98 (±0.01)
**Class 3**	92 (±0.06)	98 (±0.03)	91 (±0.05)	96 (±0.02)
**Class 4**	93 (±0.06)	98 (±0.02)	90 (±0.06)	97 (±0.03)
**Overall mean (±SD)**	**93 **(±0.01)	**98 **(±0.01)	**93 **(±0.02)	**97 **(±0.01)

**Table 4:** Sensitivity (%) and specificity (%) for each class recorded from Ch 2 and Ch 4 from amputee subject using WMD method

**Class**	**Channel 2**		**Channel 4**	
	**Sensitivity (%)**	**Specificity (%)**	**Sensitivity (%)**	**Specificity (%)**
**Class 1**	90	95	81	80
**Class 2**	82	81	80	79
**Class 3**	79	80	84	81
**Class 4**	89	85	93	92
**Overall mean (±SD)**	**85** (**±**0.05)	**85** (**±**0.06)	**85** (**±**0.05)	**83** (**±**0.06)

### Accuracy of identifying sEMG of able bodied participants

From Table 1, the overall accuracy of the detection of flexion of four classes of fingers (digits 2 to 5) using WMD was found to be 93.41(± 1.45)% when sEMG was recorded from the distal end (experiment 1) of the FDS muscle. When sEMG was recorded from the proximal end (experiment 2) of the FDS muscle, there was only a small variation (1% decrease) in the overall accuracy (92.4 ± 3.23)%.

### Accuracy of identifying sEMG of amputee

The accuracy of identification of movements from the amputee is tabulated in Table 2. The results show that the average accuracy of the detection of flexion of four classes of fingers (digits 2 to 5) performed based on the bilateral learning was found to be 81.87 (± 13.54)% from sEMG electrode location 2 (Figure 1b). The average accuracy was found to be 74.59 (± 12.52)% from the sEMG electrode location 4. The classifier did not converge for recordings from the electrode locations 1 and 3 and hence has not been reported. This demonstrated that the electrode location had a significant effect on the system accuracy for the amputee subject. For further analysis, electrode location 2 was considered.

### Sensitivity and specificity analysis

From Table 3, the sensitivity of the results for able bodied subjects was 94% while the average specificity of all classes was ~ 97% for experiment 1 and ~ 93% for experiment 2. The results indicate low Type I and Type II error. From Table 4, the average specificity was 85% for channel 2 and 84.5% for channel 4, while the average sensitivity was 85% for channel 2 and 83% for channel 4.

## Discussion

The results indicate that WMD sEMG signal analysis, which is based on the volume conduction properties of the tissues, can accurately identify individual finger actions. Based on the volume conduction model reported by [26,27], the muscle tissue forms the major portion of the volume conductor. This is an anisotropic conducting medium, having different conductivities along different axes, relatively higher conductivity in the longitudinal direction and slower in the transverse direction. Therefore electric potential travelling in transverse direction of a muscle is attenuated more rapidly as compared to longitudinal direction [26,27]. Thus the action potential originating from co-located muscle fibres would have similar magnitude and the assumption is that the muscles of the forearm have relatively small cross-sectional area. The magnitude of the singularities of the signal is inversely proportional to the distance between the electrodes and the muscle, while the density of the singularities corresponds to the relative number of motor unit action potentials (MUAP), thus corresponding to the strength of muscle contraction.

In this method, sEMG was represented by the wavelet maxima and these were then grouped based on their magnitude into four groups corresponding to the four finger actions. The average magnitude and density of each of the four groups for a time-window of 300 milliseconds [4,13,16] were the input to a support vector machine that was trained to associate these with the finger action. This time window is the permissible delay for real time operation of the system. The method was also tested for the 200 ms (< 300 ms) and no significant change in the classification accuracy was observed.

Two sets of experiments with the electrode placement distinctly different between the two experiments were conducted to determine the reproducibility of the technique. The classification accuracy for able-bodied subjects was in the range between 89% and 96%, while for the trans-radial amputee was between 62% and 96%. The lower accuracy for the trans-radial amputee may be due to number of reasons such as disuse the muscles or damage to the muscle [22], or movement of the insertion point, which makes it difficult to identify suitable location for electrode placement.

The recognition accuracy shows that this technique is significantly better compared with the technique reported by Momen et al. (56 ± 13%) [13], even though the proposed technique has used single channel while Momen et al. [13] have used two channels. The other significance is that this method has identified generic finger actions, which makes this suitable for controlling each finger of a prosthetic hand, while Momen et al. [13] and other researchers [10,11,14,20] have considered user defined actions. The other strength of this technique is that it is repeatable and not sensitive to differences in electrode placement.

The system requires supervised training, while the classification phase is suitable for being automated without manual supervision. The technique could be applied for prosthetic hand control or for human computer interface.

## Conclusion

In this study, we have investigated and reported a novel method to estimate the relative strength of contraction of different muscles to identify individual finger flexions. This method is based on the volume transmission properties of action potentials in body tissues [16], leading to the MUAP of different muscles having different amplitudes. During the low level of muscle contraction such as isometric finger flexion, MUAP are sparse and thus the measure of density of MUAP in terms of the amplitude leads to a measure of strength of contraction of different muscles.

Wavelet maxima detection is a suitable technique to identify the local peaks in the sEMG. Identifying the range of the amplitude of peaks using clustering techniques using the wavelet maxima density (WMD) is an indicator of the muscle activity of different muscles. This technique uses WMD as the set of features that can be classified using twin support vector machine (TSVM) to determine the associated finger flexion. The advantage of the TSVM over other classifier techniques is that it is suitable for unbalanced data sets.

The results show that using WMD, single channel EMG is suitable for accurately identifying individual finger flexion. This technique was tested for able bodied and trans-radial amputee subject and also for variations in the placement of the electrodes. This work has demonstrated that it is possible to accurately identify the intent of individual finger flexion using single channel EMG recorded from the surface of the stump of a transradial amputee patient.

## Competing interests

The authors declare that they have no competing interests.

## Authors’ contributions

DKK has designed the experiment, discussed and developed the underlying concepts for the technique and finalised the manuscript. SPA has also designed and conducted the experiments on the amputee subject, performed data analysis and written part of the manuscript. VPS has conducted the experiment with able bodies and developed the wavelet based signal processing technique. All authors read and approved the final manuscript.
